# Framing effects in value-directed remembering

**DOI:** 10.3758/s13421-022-01317-y

**Published:** 2022-04-29

**Authors:** Dillon H. Murphy, Barbara J. Knowlton

**Affiliations:** Department of Psychology, University of California Los Angeles, Los Angeles, CA 90095 USA

**Keywords:** Value-directed remembering, Framing, Gains, Losses, Metacognition

## Abstract

Changing how an issue is framed can influence both decision-making and metacognition, but framing a memory task in terms of gains and losses could also impact how learners prioritize information according to its value or importance. We investigated how framing task instructions and feedback in terms of gains and losses influences learners’ ability to selectively remember valuable information at the expense of low-value information. Specifically, we presented learners with to-be-remembered words paired with point values and either told participants how many points they scored (the sum of the values of recalled words) or lost (the sum of the values of not-recalled words) on each list, with participants’ goal being to maximize their scores or minimize their losses, respectively. Overall, participants were more selective for high-value words when their goals were framed in terms of point gains compared with when their goals were framed in terms of losses, and learners’ metacognitive predictions of performance (JOLs) generally mapped onto this trend. Thus, framing in terms of losses for forgetting can reduce memory selectivity, perhaps because even small losses are salient, indicating that framing effects are not limited to decision-making but can influence memory and metacognitive processes as well.

In everyday life, we are exposed to far more information than can be remembered. For example, the internet provides virtually endless amounts of information; social media floods our phones with pictures, messages, and updates from friends; and students often have several book chapters, lectures, and homework assignments to review before exams. Since this is usually too much to remember, we tend to focus on and selectively remember the most important information. Similarly, when overwhelmed with information in the laboratory, participants tend to focus on and direct resources toward high-value information to maximize the likelihood that this information will be effectively encoded and later recalled (Ariel et al., 2009; Castel et al., 2012), a type of selective memory crucial for maximizing memory utility.

To measure this form of selective memory, value-directed remembering tasks present participants with to-be-remembered items paired with various point values counting toward their score if recalled. In these tasks, participants tend to use value to guide the encoding and retrieval processes by best recalling valuable information (Castel et al., 2002; Elliott et al., 2020; Hennessee et al., 2019; Murphy & Castel, 2022a; Stefanidi et al., 2018; see Knowlton & Castel, 2021; Madan, 2017, for a review). This selectivity for valuable information often increases as the learner gains task experience (Castel, 2008; Castel et al., 2012; McGillivray & Castel, 2011) and learners are generally metacognitively aware of their selectivity (see Murphy, Agadzhanyan, et al., 2021a; Murphy, Huckins, et al., 2021b).

To strategically remember valuable information, often at the expense of low-value information, an understanding of how one’s memory works (metamemory) is crucial. Metamemory, specifically metacognition, involves the awareness and understanding of one's memory processes (see Nelson, 1996; Nelson & Narens, 1990). When evaluating the likelihood of remembering information, people engage in metacognitive monitoring, a process of assessing future memory performance. For example, many studies have solicited judgments of learning (JOLs), whereby participants indicate the likelihood of remembering information (see Rhodes, 2016, for a review). These assessments typically occur during the encoding phase such that judgments are made immediately after an item is studied. Thus, monitoring assessments are often informed by the cues available during learning (i.e., intrinsic, extrinsic, mnemonic cues; see Koriat, 1997).

When choosing what information to study, learners’ sensitivity to an item’s value or importance may be influenced by the framing of the tasks’ demands. The framing effect involves influenced decision-making according to how equivalent information is presented (i.e., based on what features are emphasized), and many studies have illustrated the impact of framing effects on people’s social and economic decisions (see Kühberger, 1997; Steiger & Kühberger, 2018, for a review). Specifically, several different mechanisms can contribute to the framing effect based on what is being framed and the type of choice faced (Levin et al., 1998). For example, in some cases, some attributes of an object may be framed negatively or positively such as whether a meat product is labeled as 90% lean or 10% fat, and these framings can affect peoples’ feelings of attraction toward the product (e.g., Levin, 1987). In other work, goal-directed behavior can be influenced by a message that either stresses positive aspects of achieving a goal or negative aspects of not achieving a goal (e.g., Banks et al., 1995). Lastly, framing outcomes in terms of gains (e.g., number of lives saved) or losses (e.g., number of lives lost) can also influence decision-making (e.g., Tversky & Kahneman, 1981).

In addition to influencing attitudes, goals, and decision-making, framing effects can also influence metacognitive judgments. For example, when metacognitive monitoring judgments are framed in terms of forgetting rather than remembering (i.e., judgments of the likelihood of *forgetting* a word versus judgments of the likelihood of *remembering* a word), confidence in memory performance tends to decrease (e.g., Finn, 2008). Thus, since changing how an issue is presented can also influence both decision-making and metacognition, framing a memory task in terms of gains and losses could impact how learners prioritize information according to its value or importance.

Similar to framing effects, loss aversion is a basic principle of decision-making whereby losses are experienced more strongly than gains of a similar degree (see Hastie, 2001; Kahneman & Tversky, 1979; Thaler, 1999; Tversky, 1994; Tversky & Kahneman, 1991, 1992). As such, people are *risk-averse* for gains (they do not want to risk losing a possible gain) but *risk-seeking* for losses (losses loom larger than gains; Tversky & Kahneman, 1981; see also Whitney et al., 2008). Thus, the same situation may feel worse when framed in terms of losses than when framed in terms of gains. For example, people may work relatively harder to avoid a loss incurred due to forgetting than to incur a gain through remembering.

When attempting to remember important information (i.e., a child’s allergies or your passport when packing for a vacation), learners should prioritize memory for this information to minimize the negative consequences of forgetting. *Responsible remembering* is a form of adaptive memory and refers to how our memory functions to prioritize important information or information with negative consequences if forgotten (see Murphy & Castel, 2020, 2021a, 2021b, 2022b; Murphy et al., 2022), and responsible remembering mechanisms allow for the selective encoding of valuable information to maximize memory utility and prevent the forgetting of valuable information. Applied to framing effects, learners may differentially engage responsible remembering mechanisms to either seek the gains of remembering important information or avoid the consequences (i.e., losses) for forgetting valuable information based on the framing of their goals. Specifically, when seeking gains, learners may be more conservative in terms of how much they attempt to remember and prioritize memory for high-value words. In contrast, when evading losses, learners may be inclined to try to remember more information to avoid the costs of forgetting, but this may come at the expense of memory selectivity.

## The current study

In the current study, we investigated how framing task instructions and feedback in terms of gains and losses influences how learners selectively remember valuable information at the expense of low-value information as well as the potential metacognitive awareness of these effects. Specifically, we presented participants with words paired with point values and either told participants how many points they scored (the sum of the values of recalled words) or how many points they lost (the sum of the values of not-recalled words) on each list. Since previous work indicates that people tend to be risk-averse in the face of gains and risk-seeking in the face of losses, we expected participants to be less selective for high-value words when their task goals are framed in terms of losses because participants will try to avoid small as well as large losses rather than focusing on just high-value words. We also expected that metacognitive monitoring judgments and control decisions regarding study time would reflect this greater sensitivity to losses.

## Experiment 1

In Experiment 1, we presented participants with lists of words to remember for a later test (using a fixed study schedule) with each word accompanied by a number indicating how much the word is worth on a subsequent memory test. However, some participants were told that for every word they recalled, that word’s point value would be added toward their task score, with participants’ goal being to maximize their score. When receiving feedback after each list’s recall test, these participants were told how many points they earned out of how many points they could have earned. In contrast, rather than framing participants’ goals in terms of gains, other participants’ instructions and feedback were framed in terms of losses. Specifically, these participants were told that they would lose points for every word they forget, with participants’ goal being to minimize their losses. When receiving feedback at the end of each list’s recall test, these participants were told how many points they lost out of how many possible points they could have lost. Furthermore, all participants were asked to predict the likelihood of remembering each word (JOL). We expected participants to be less selective for high-value words when task goals were framed in terms of losses and for this to also be reflected in their metacognitive monitoring judgments.

### Method

#### Participants

After exclusions, participants were 107 undergraduate students (age range: 18–48; *M*_age_ = 20.53, *SD*_age_ = 3.79) recruited from the University of California Los Angeles (UCLA) Human Subjects Pool. Participants were tested online (in a place of their own choosing) and received course credit for their participation (see Anwyl-Irvine et al., 2021, for an examination of the accuracy and precision of online data; see Greene & Naveh-Benjamin, 2022, for a discussion of the advantages and disadvantages of online data collection). Participants were excluded from analysis if they admitted to cheating (e.g., writing down answers) in a posttask questionnaire (they were told they would still receive credit if they cheated). This exclusion process resulted in three exclusions. In Experiments 1 and 2, we aimed to collect around 100 participants. The sample size was selected based on prior research and the expectation of detecting a medium effect size in terms of the effect of value on probability or recall. With this sample size, we had an 80% chance of detecting a *medium* (Cohen’s *d* = .55) effect between conditions (framing: gains, losses).

#### Materials and procedure

**Fig. 1:**
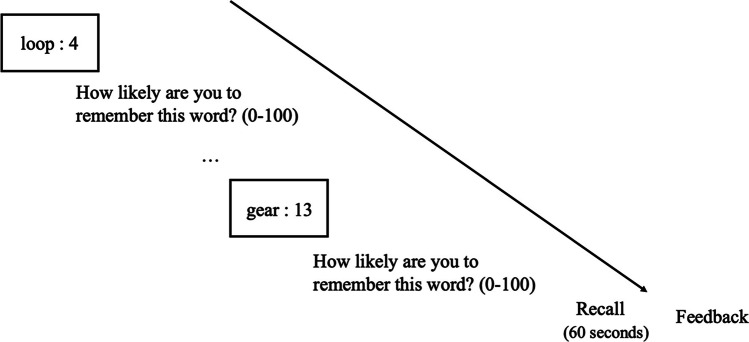
The general procedure for each list in Experiments 1 and 2

The general procedure used in Experiments 1 and 2 is shown in Fig. 1. To participate, participants were given a link to complete the online study that took them to a webpage welcoming them to the experiment. They were thanked for agreeing to participate in the study and asked to use either Chrome or Firefox. Participants then clicked a button to advance to the task instructions where they were told that they would be presented with lists of words with each list containing 20 different words. Participants were also told that each word would be presented for 3 seconds each and that after each list was presented they would have 1 minute to recall the words from just that list (i.e., not previous lists).

Next, participants were told that each word in each list would be paired with a unique, randomly assigned value between 1 and 20 indicating how much the word was “worth.” However, some participants’ instructions about the point values were framed in terms of gains (*n* = 53) while other participants’ instructions about the point values were framed in terms of losses (*n* = 54). Specifically, when participants’ instructions were framed in terms of gains, they were told that “Each word will be accompanied by an associated number. This number indicates how many points the word is worth. For example, if the word ‘apple’ appears with a 5 next to it and you remember ‘apple’ during the test, then you will receive 5 points. The numbers will range from 1 to 20. After each test, you will be told your score for that list. Do your best to maximize your score.” In contrast, when participants’ instructions were framed in terms of losses, they were told that “Each word will be accompanied by an associated number. This number indicates how many points the word is worth. For example, if the word ‘apple’ appears with a 5 next to it and you forget ‘apple’ during the test, then you will lose 5 points. The numbers will range from 1 to 20. After each test, you will be told how many points you lost for that list. Do your best to minimize your losses.”

Immediately following the recall period, participants were given feedback on their performance for the list but were not given feedback about specific items (we provided participants with aggregate feedback because providing participants with the total number of points they scored or lost could have a greater influence on selective memory processes than simply confirming the correctness of each item in participants’ output). However, when participants’ instructions were framed in terms of gains, their feedback was phrased in terms of how many points they scored out of how many points they possibly could have scored (i.e., “You got 100 out of 210 points”). In contrast, when participants’ instructions were framed in terms of losses, their feedback was phrased in terms of how many points they lost out of how many points they possibly could have lost (i.e., “You lost 110 out of 210 points”).^1^

Each point value was used only once within each list and the order of the point values within lists was randomized. After each word was presented, participants were asked to estimate the likelihood of correctly recalling it on a later test (JOL). Participants answered with a number between 0 and 100, with 0 meaning they definitely would not remember the word and 100 meaning they definitely would remember the word. Participants were given as much time as they needed to make their judgments. After the presentation of all 20 word–number pairs in each list, participants were given a 1-minute free recall test in which they had to recall as many words as they could from the list (they did not need to recall the point values). This was repeated for six study–test trials.

### Results

To examine differences in JOLs, recall, and selectivity for valuable information, we used Jamovi to compute multilevel models (MLMs) where we treated the data as hierarchical or clustered (i.e., multilevel) with items nested within individual participants. Since recall at the item level was binary (correct or incorrect), we conducted logistic MLMs in our examination of recall. In these analyses, the regression coefficients are given as logit units (i.e., the log odds of correct recall). We report exponential betas (e^B^), and their 95% confidence intervals (CI_95%_), which give the coefficient as an odds ratio (i.e., the odds of correctly recalling a word divided by the odds of not recalling a word). Thus, e^B^ can be interpreted as the extent to which the odds of recalling a word changed. Specifically, values greater than 1 represent an increased likelihood of recall while values less than 1 represent a decreased likelihood of recall.

**Fig. 2:**
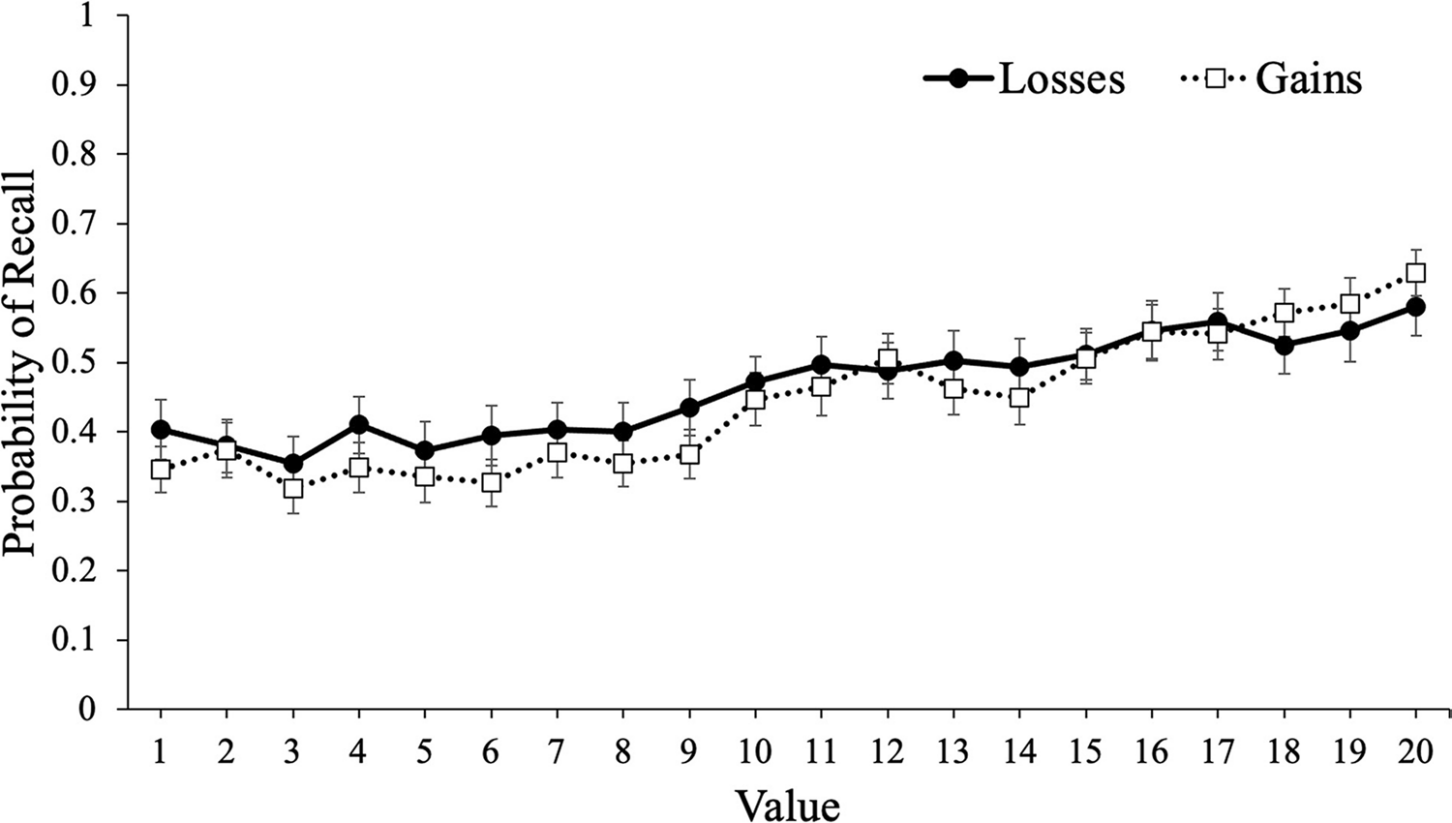
Probability of recall as a function of framing and word value in Experiment 1. Error bars reflect the standard error of the mean

To examine recall, we conducted a logistic MLM with item-level recall modeled as a function of value with framing (gains, losses) as a between-subjects factor. Results revealed that value significantly predicted recall, e^B^ = 1.07, CI_95%_ [1.06, 1.07], *z* = 18.71, *p* < .001, such that high-value words were better recalled than low-value words. However, framing did not significantly predict recall, e^B^ = 1.08, CI_95%_ [.74, 1.58], *z* = .40, *p* = .692, such that participants whose goals were framed in terms of gains (*M* = .44, *SD* = .17) recalled a similar proportion of words as participants whose goals were framed in terms of losses (*M* = .46, *SD* = .22). Critically, value interacted with framing, e^B^ = .98, CI_95%_ [.97, 1.00], *z* = 2.46, *p* = .014, such that value was a stronger predictor of recall for participants whose goal was framed in terms of gains (e^B^ = 1.08) than when goals were framed in terms of losses (e^B^ = 1.06; see Fig. 2).

**Fig. 3:**
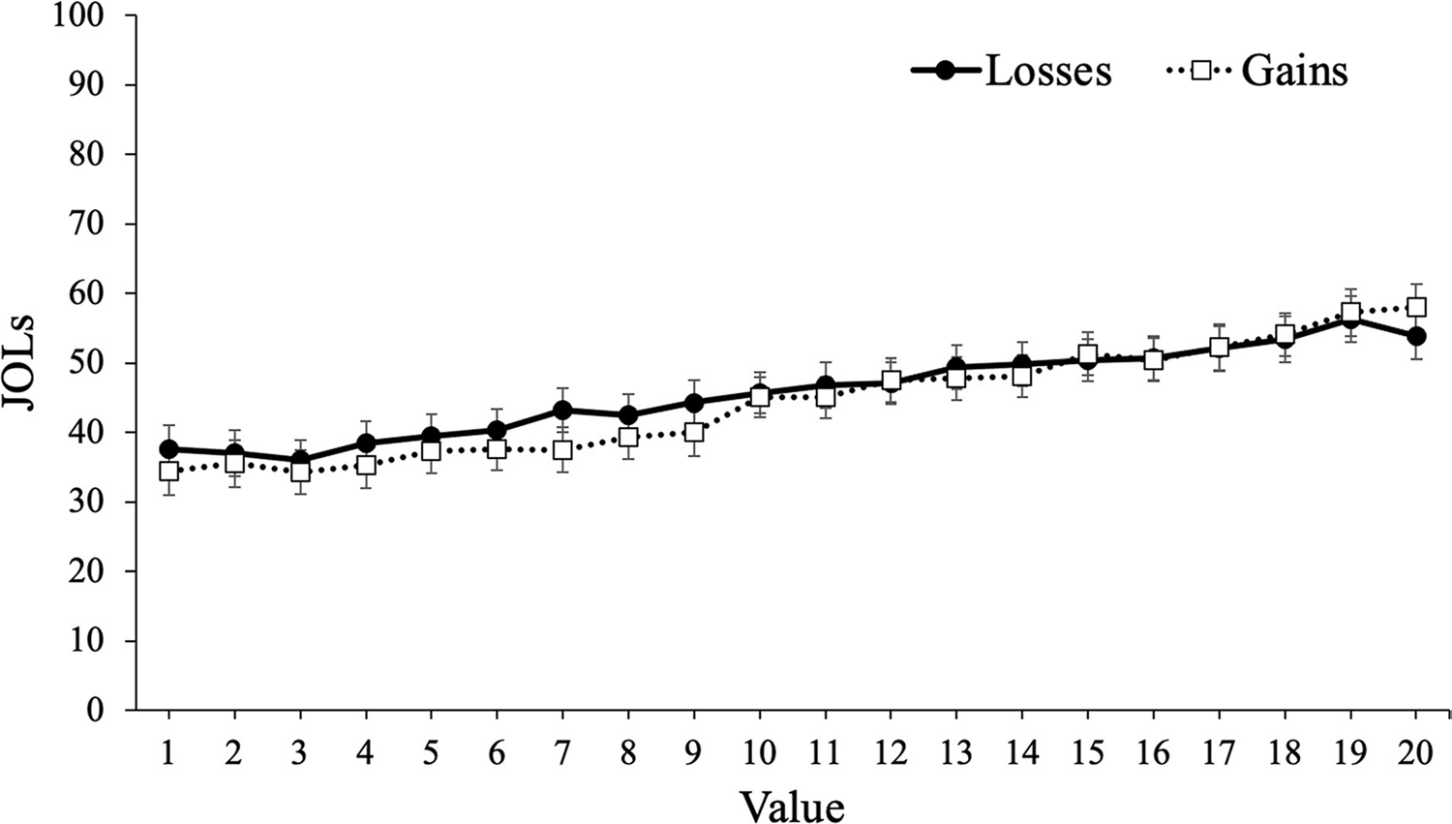
Judgments of learning (JOLs) as a function of framing and word value in Experiment 1. Error bars reflect the standard error of the mean

To examine participants’ JOLs, we conducted a mixed MLM with item-level JOLs modeled as a function of value with framing (gains, losses) as a between-subjects factor. Results revealed that value significantly predicted JOLs, *t*(12726) = 31.50, *p* < .001, such that participants expected to better remember high-value words. However, framing did not predict JOLs, *t*(105) = .35, *p* = .731, such that participants with goals framed in terms of gains (*M* = 44.44, *SD* = 19.57) expected similar recall rates as participants with goals framed in terms of losses (*M* = 45.75, *SD* = 19.84). Critically, value interacted with framing, *t*(12726) = 3.68, *p* < .001, such that value was a stronger predictor of JOLs for participants whose goals were framed in terms of gains (coefficient estimate: 1.31) than participants whose goals were framed in terms of losses (coefficient estimate: 1.04; see Fig. 3).

We also examined whether there was greater metacognitive accuracy (relationship between predictions and recall; see Rhodes, 2016) under one of the framing conditions. Specifically, we conducted a logistic MLM with item-level recall modeled as a function of JOLs with framing (gains, losses) as a between-subjects factor (see Murayama et al., 2014). Results revealed that JOLs significantly predicted recall, e^B^ = 1.03, CI_95%_ [1.03, 1.03], *z* = 32.97, *p* < .001, such that words given higher JOLs were better recalled than words given low JOLs. However, framing did not significantly predict JOLs, e^B^ = 1.04, CI_95%_ [.69, 1.55], *z* = .18, *p* = .858, and JOLs did not interact with framing, e^B^ = 1.00, CI_95%_ [1.00, 1.01], *z* = .74, *p* = .461.

#### Discussion

In Experiment 1, we presented participants with words to remember for a later test with the values accompanying each word either framed in terms of gains or losses. Despite no group differences in recall, results revealed that value was a better predictor of recall (as well as participants’ predictions of performance) when goals were framed in terms of gains. Specifically, participants were more selective for high-value information when their goals were framed in terms of maximizing their score compared with minimizing their losses. Thus, while the framing of information can influence decision-making, selective memory can also be affected by framing, and participants were generally metacognitively aware of this effect.

## Experiment 2

In Experiment 2, we were interested in how allowing learners to self-regulate their study time influences the potential framing effects on memory selectivity. In a similar design to Experiment 1, participants again studied lists of words paired with point values and after each list, participants were either given the sum of the values of the words they recalled (gains) or forgot (losses). We again expected participants seeking gains to be more selective and for this to be reflected in their metacognitive monitoring and control decisions.

### Method

#### Participants

After exclusions, participants were 107 undergraduate students (age range: 18–41; *M*_age_ = 20.43, *SD*_age_ = 2.90) recruited from the UCLA Human Subjects Pool. Participants were tested online and received course credit for their participation. Participants were excluded from analysis if they admitted to cheating (e.g., writing down answers) in a posttask questionnaire (they were told they would still receive credit if they cheated). This exclusion process resulted in seven exclusions. Participants in Experiment 2 did not participate in Experiment 1. With this sample size, we had an 80% chance of detecting a *medium* (Cohen’s *d* = .55) effect between conditions (framing: gains, losses).

#### Materials and procedure

The materials and procedure in Experiment 2 were similar to Experiment 1. However, participants were told that they could study each word for as long as they liked, with a maximum study time of 10 seconds per word. Specifically, in the study phase, participants clicked a button on the screen to advance to the next word when they were ready, or the task automatically advanced to the next word if participants did not click the button within 10 seconds. Again, participants were either given instructions framed in terms of gains (*n* = 54) or losses (*n* = 53).

### Results

**Fig. 4:**
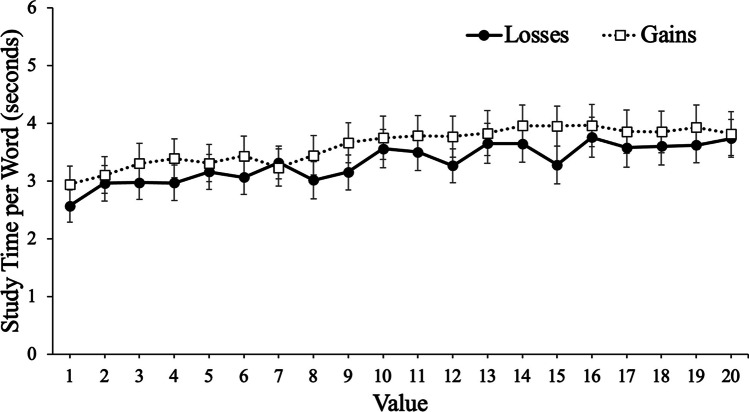
Study time per word (seconds) as a function of framing and word value in Experiment 2. Error bars reflect the standard error of the mean

To examine participants’ study time, we conducted a mixed MLM with item-level study time modeled as a function of value with framing (gains, losses) as a between-subjects factor. Results revealed that value significantly predicted study time, *t*(12731) = 14.45, *p* < .001, such that participants spent more time studying high-value words. However, framing did not predict study time, *t*(105) = .67, *p* = .504, such that participants with goals framed in terms of gains (*M* = 3.61 s, *SD* = 2.44) studied each word for a similar duration (seconds) as participants with goals framed in terms of losses (*M* = 3.32 s, *SD* = 2.10). Furthermore, value did not interact with framing, *t*(12731) = .12, *p* = .908, such that value was a similar predictor of study time regardless of the framing of participants’ goals (see Fig. 4).

**Fig. 5:**
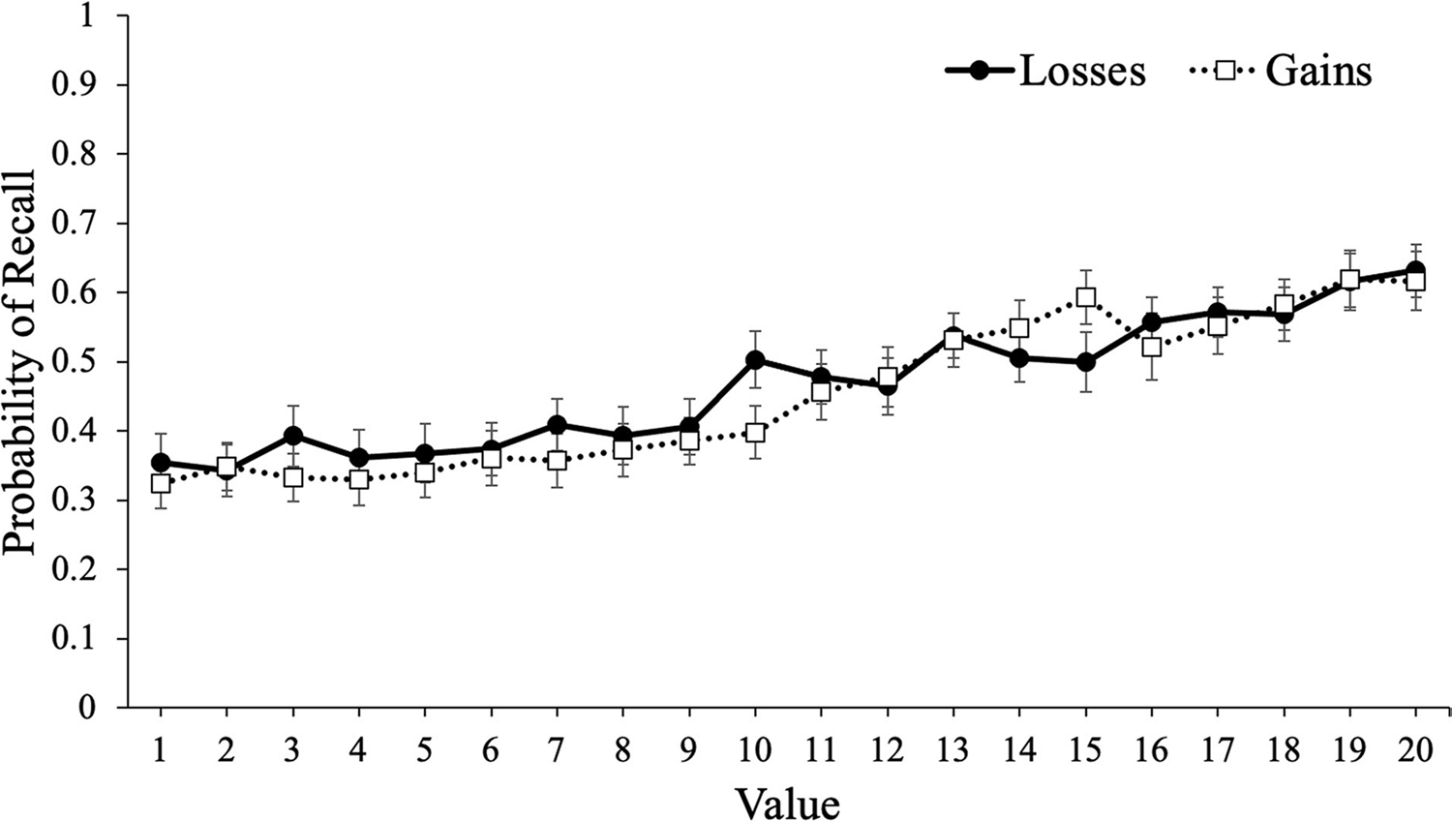
Probability of recall as a function of framing and word value in Experiment 2. Error bars reflect the standard error of the mean

To examine recall, we conducted a logistic MLM with item-level recall modeled as a function of value with framing (gains, losses) as a between-subjects factor. Results revealed that value significantly predicted recall, e^B^ = 1.08, CI_95%_ [1.08, 1.09], *z* = 22.84, *p* < .001, such that high-value words were better recalled than low-value words. However, framing did not significantly predict recall, e^B^ = 1.06, CI_95%_ [.72, 1.56], *z* = .31, *p* = .759, such that participants whose goals were framed in terms of gains (*M* = .45, *SD* = .19) recalled a similar proportion of words as participants whose goals were framed in terms of losses (*M* = .47, *SD* = .20). Finally, there was only a weak trend for an interaction between value and framing, e^B^ = .99, CI_95%_ [.98, 1.00], *z* = 1.71, *p* = .088. While the recall data in Experiment 2 showed a similar pattern to the data of Experiment 1, particularly for low-value items, there was no significant difference between the framing conditions in terms of how value was predictive of recall (see Fig. 5).

**Fig. 6:**
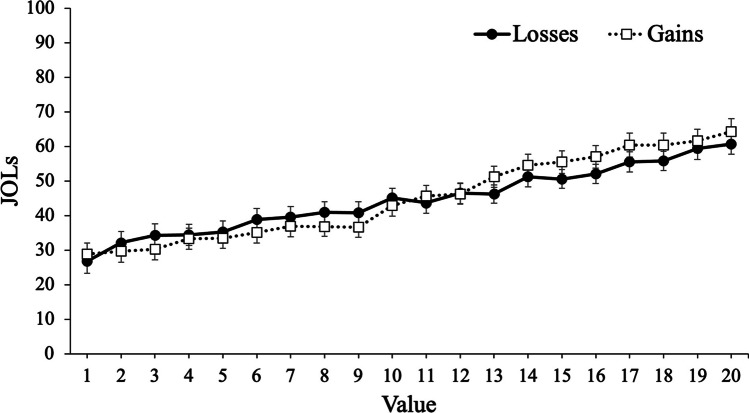
Judgments of learning (JOLs) as a function of framing and word value in Experiment 2. Error bars reflect the standard error of the mean

To examine participants’ JOLs, we conducted a mixed MLM with item-level JOLs modeled as a function of value with framing (gains, losses) as a between-subjects factor. Results revealed that value significantly predicted JOLs, *t*(12727) = 49.42, *p* < .001, such that participants expected to better remember high-value words. Framing did not predict JOLs, *t*(105) = .16, *p* = .876, such that participants with goals framed in terms of gains (*M* = 45.08, *SD* = 18.64) expected similar recall rates as participants with goals framed in terms of losses (*M* = 44.52, *SD* = 18.25). Critically, as in Experiment 1, value interacted with framing, *t*(12727) = 5.87, *p* < .001, such that value was a stronger predictor of JOLs for participants whose goals were framed in terms of gains (coefficient estimate: 2.02) than participants whose goals were framed in terms of losses (coefficient estimate: 1.59; see Fig. 6).

Finally, we again examined whether there was greater metacognitive accuracy under one of the framing conditions. Specifically, we conducted a logistic MLM with item-level recall modeled as a function of JOLs with framing (gains, losses) as a between-subjects factor. Results revealed that JOLs significantly predicted recall, e^B^ = 1.03, CI_95%_ [1.03, 1.03], *z* = 33.92, *p* < .001, such that words given higher JOLs were better recalled than words given low JOLs. However, framing did not significantly predict JOLs, e^B^ = 1.09, CI_95%_ [.74, 1.61], *z* = .43, *p* = .670, and JOLs did not interact with framing, e^B^ = 1.00, CI_95%_ [1.00, 1.01], *z* = 1.39, *p* = .166.

### Discussion

In Experiment 2, we allowed participants to self-regulate their study time of to-be-remembered words paired with point values. However, in contrast to Experiment 1, there were no significant differences in selectivity as a function of whether participants’ goals were phrased in terms of gains or losses. Despite no group differences in selectivity, participants whose goals were phrased in terms of gains expected to be more selective (as indicated by their JOLs). Specifically, these participants expected to better remember high-value words than low-value words compared with participants whose goals were framed in terms of losses, but there were no differences as a function of framing in terms of how value informed the metacognitive control decision of how long to study each item. Thus, the influence of framing effects on selectivity may be mitigated when learners can control the study phase.

## Experiment 3

In Experiment 1, under fixed encoding conditions, framing learners’ goals in terms of gains enhanced selectivity relative to learners whose goals were framed in terms of losses, but this effect did not reach significance in Experiment 2 when the encoding phase was self-paced. In Experiment 3, we aimed to replicate the findings from Experiments 1 and 2 as well as directly compare the effects of framing on memory selectivity under fixed and self-paced encoding conditions. Additionally, we did not solicit JOLs to evaluate whether the effects of framing on memory selectivity persist when not explicitly monitoring learning.

### Method

#### Participants

After exclusions, participants were 354 undergraduate students (age range: 18–50; *M*_age_ = 20.75, *SD*_age_ = 3.43) recruited from the UCLA Human Subjects Pool. Participants were tested online and received course credit for their participation. Participants were excluded from analysis if they admitted to cheating (e.g., writing down answers) in a posttask questionnaire (they were told they would still receive credit if they cheated). This exclusion process resulted in 21 exclusions. Participants in Experiment 3 did not participate in Experiments 1 or 2. Since the effect of framing was small in Experiment 1, in Experiment 3 we greatly increased our sample size. With this sample size, we had an 80% chance of detecting a *medium* (Cohen’s *d* = .30) effect between conditions (framing: gains, losses).

#### Materials and procedure

The materials and procedure in Experiment 3 were similar to Experiments 1 and 2. However, participants did not provide JOLs. Study time was either fixed (3 seconds; *n* = 176) or self-paced (*n* = 178) and participants were either given instructions framed in terms of gains (*n* = 176) or losses (*n* = 178).

### Results

**Fig. 7:**
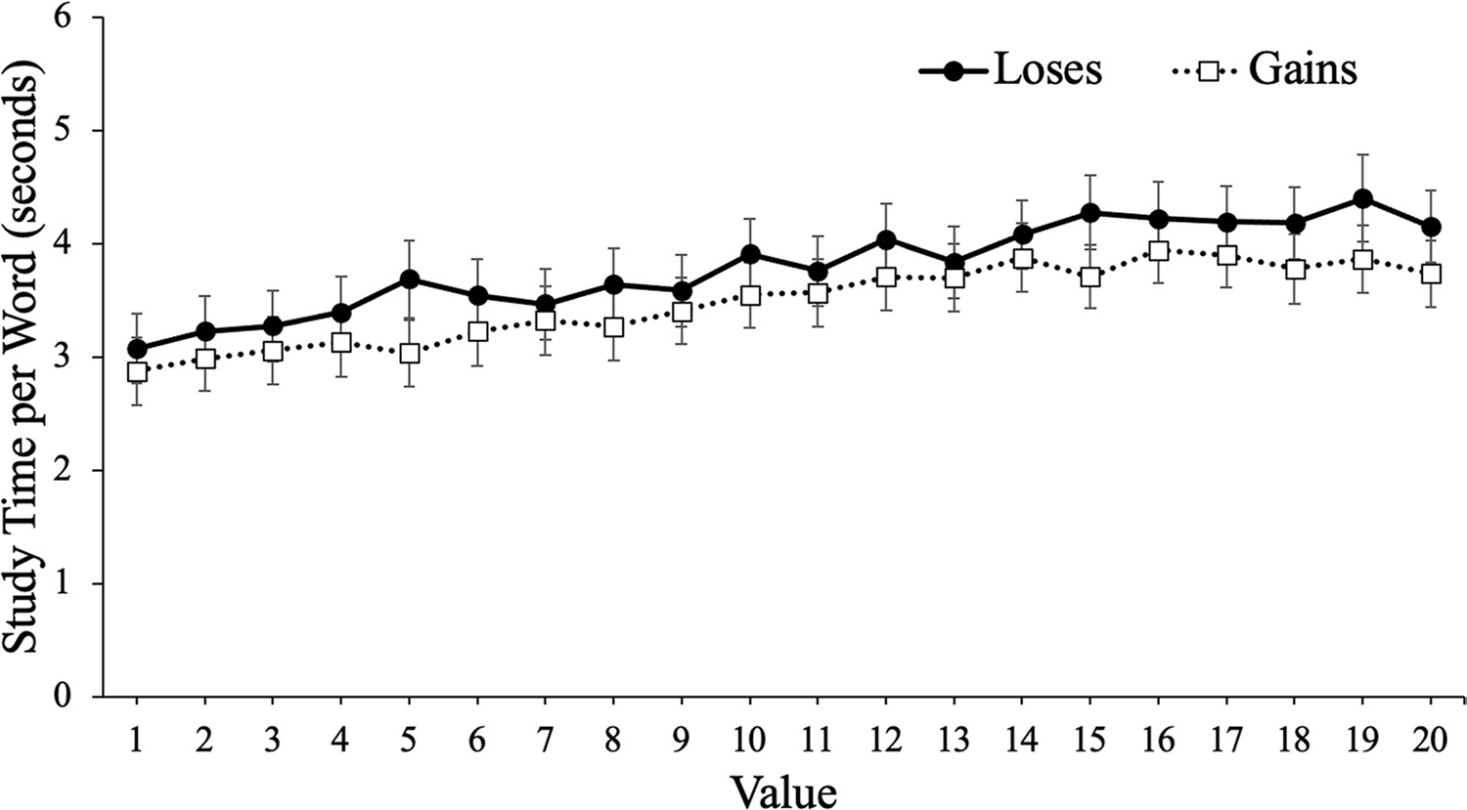
Study time per word (seconds) as a function of framing and word value in Experiment 3. Error bars reflect the standard error of the mean

To examine participants’ allocation of study time, we conducted a mixed MLM with item-level study time modeled as a function of value with framing (gains, losses) as a between-subjects factor. Results revealed that value significantly predicted study time, *t*(21180) = 20.72, *p* < .001, such that participants spent more time studying high-value words. However, framing did not predict study time, *t*(176) = .77, *p* = .443, such that participants with goals framed in terms of gains (*M* = 3.48s, *SD* = 2.66) studied each word for a similar duration as participants with goals framed in terms of losses (*M* = 3.80s, *SD* = 2.83). Furthermore, value did not interact with framing, *t*(21180) = 1.34, *p* = .181, such that value was a similar predictor of study time regardless of the framing of participants’ goals (see Fig. 7).

**Fig. 8:**
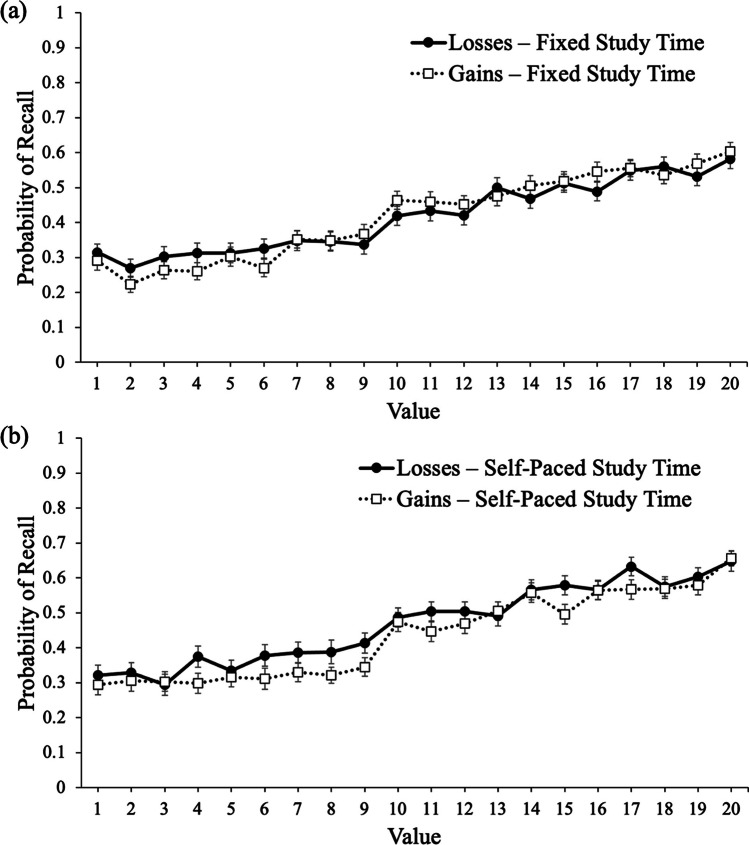
Probability of recall as a function of framing and word value when study time was fixed (**a**) and self-paced (**b**) in Experiment 3. Error bars reflect the standard error of the mean

To examine recall, we conducted a logistic MLM with item-level recall modeled as a function of value with framing (gains, losses) and study schedule (fixed, self-paced) as between-subjects factors. Results revealed that value significantly predicted recall, e^B^ = 1.09, CI_95%_ [1.09, 1.10], *z* = 46.53, *p* < .001, such that high-value words were better recalled than low-value words. However, framing did not significantly predict recall, e^B^ = 1.08, CI_95%_ [.91, 1.27], *z* = .88, *p* = .378, such that participants whose goals were framed in terms of gains (*M* = .43, *SD* = .15) recalled a similar proportion of words as participants whose goals were framed in terms of losses (*M* = .44, *SD* = .18). Study schedule predicted recall, e^B^ = 1.18, CI_95%_ [1.00, 1.39], *z* = 1.99, *p* = .046, such that participants who self-paced their study time recalled a greater proportion of words (*M* = .45, *SD* = .18) than participants who studied each word for 3 seconds (*M* = .42, *SD* = .15). Framing and study schedule did not interact, e^B^ = 1.17, CI_95%_ [.84, 1.63], *z* = .95, *p* = .344, but value interacted with framing, e^B^ = .99, CI_95%_ [.98, 1.00], *z* = −2.39, *p* = .017, such that value was a stronger predictor of recall for participants whose goal was framed in terms of gains (e^B^ = 1.10) than participants whose goal was framed in terms of losses (e^B^ = 1.09). Value did not interact with study schedule, e^B^ = 1.01, CI_95%_ [1.00, 1.01], *z* = 1.63, *p* = .103, and there was not a significant three-way interaction between value, framing, and study schedule, e^B^ = 1.01, CI_95%_ [1.00, 1.03], *z* = 1.68, *p* = .093 (see Fig. 8).

### Discussion

In Experiment 3, results largely replicated Experiments 1 and 2. Specifically, participants whose goals were framed in terms of gains were more selective than participants whose goals were framed in terms of losses. However, this did not differ as a function of study schedule (fixed or self-paced). Thus, the effects of framing on memory selectivity may not be driven by metacognitive monitoring judgments (participants did not make JOLs in Experiment 3) or control decisions.

## General discussion

We are often presented with more information than we can remember but how that information is framed may influence memory. The framing effect refers to instances where equivalent information presented in different ways (i.e., gains versus losses) can influence behavior (see Kühberger, 1997; Steiger & Kühberger, 2018, for a review). Specifically, people’s judgments and decisions about an identical situation can be influenced by positive or negative framing (Tversky & Kahneman, 1981). In the context of memory, it is often of adaptive benefit to remember important information at the expense of less important information (e.g., Murphy & Castel, 2020, 2021a, 2021b, 2022b; Murphy et al., 2022) so learners should be motivated to maximize gains while also minimizing losses. However, the phrasing of one’s goals may influence their ability to engage in this adaptive form of memory whereby learners prioritize important information.

When making decisions, loss aversion refers to a greater impact of losses than gains of a similar magnitude (see Hastie, 2001; Kahneman & Tversky, 1979; Thaler, 1999; Tversky, 1994; Tversky & Kahneman, 1991, 1992). Furthermore, when evaluating potential gains and losses, people are generally *risk-averse* for gains but *risk-seeking* for losses (Tversky & Kahneman, 1981; see also Whitney et al., 2008). Applied to selective memory, if one’s goals are phrased in terms of gains, learners may be more conservative in their study strategies by focusing more on high-value information at the expense of low-value information. In contrast, if one’s goals are phrased in terms of losses, learners may be more liberal in their attempted memory of each of the to-be-remembered words which may come at the cost of memory selectivity.

In the current study, we presented participants with to-be-remembered words paired with point values and after each list, we either informed participants how many points they scored (sum of the point values of recalled words) or how many points they lost (sum of the point values of forgotten words). Overall, participants were less selective when their goals were framed in terms of losses compared with when their goals were framed in terms of gains, and learners’ metacognitive predictions of performance (JOLs) generally mapped onto this trend. However, self-pacing study time did not significantly influence the effect of framing on memory selectivity.

When goals are framed in terms of losses, participants may allocate more cognitive resources toward low-value words than participants whose goals are framed in terms of gains. As such, to minimize their losses, these participants may engage in less efficient encoding strategies whereby they fail to prioritize important information, leading to the forgetting of high-value words. Specifically, participants attempting to maximize their gains may be more selective during the encoding phase by prioritizing high-value words at the expense of low-value words (which do relatively less to maximize their score). For example, if a learner can only remember 10 of 20 to-be-remembered words, the most efficient strategy to maximize their score would be to remember only the 10 highest valued words. However, framing goals in terms of avoiding losses may influence a learners’ ability to engage in selective memory, as even small losses are seen as important. As a result, these participants may not prioritize high-value words to the extent of a learner aiming to maximize their gains, leading to poorer memory selectivity.

In the Responsible Remembering framework of memory (Murphy & Castel, 2020), learners should be selective for valuable, important information to prevent negative consequences for forgetting. However, the present results suggest that framing in terms of negative consequences may lead to less selectivity, with more cognitive resources devoted to preventing small losses compared with those devoted to acquiring small gains. Under conditions in which study time is limited, this could lead to less efficient memory. For example, in a grading scheme in which a differential number of points is *deducted* for errors in details versus core concepts, learners may spend more time on details at the expense of more important core concepts. Framing this grading scheme differently, if differential points are *awarded* for the correct recall of details versus core concepts, learners may more effectively focus on the core information.

In the present study, losses and gains were both effective in motivating memory. However, in real-life settings, forgetting critical information may have serious consequences, such as forgetting that an individual has a food allergy or that a child needs to be picked up at school (see Middlebrooks et al., 2016, for an example of a value-directed remembering paradigm that framed goals under the context of memory for food allergies). In these settings, potential large losses for forgetting may be sufficiently salient to engage effective encoding. As such, future work may benefit from examining framing effects on selective memory in more applied settings, including the classroom. Additionally, future work should examine the effect of framing on memory using incentives rather than point values. For example, paying participants for correct recall or subtracting money from an initial payment based on memory performance may differentially affect selective memory compared with point values. Future work could also examine how framing impacts selective memory in the lab rather than online (as was done in the current experiments).

In sum, the present study demonstrated that selective memory can be enhanced when learners’ goals are framed in terms of gains compared with losses. Additionally, learners are metacognitively aware of this effect and self-pacing study time did not significantly reduce framing effects on memory selectivity. Thus, framing effects are not limited to decision-making but can influence memory and metacognitive processes as well.

## Footnotes

1 While one could conceptualize task scores as either starting at zero and going up for every recalled word (gains) or starting at 210 (the maximum score) and going down for every word not recalled, both forms of feedback involved the addition of point values. Specifically, the gains group was told how many points they received out of the number of points they could have received (their feedback was calculated as the sum of the values of recalled words) and the losses group was told how many points they lost out of the number of points they could have lost (their feedback was calculated as the sum of the values of not-recalled words).
